# Arginase impairs hypoxic pulmonary vasoconstriction in murine endotoxemia

**DOI:** 10.1186/s12931-019-1062-6

**Published:** 2019-06-03

**Authors:** Bodil Petersen, Cornelius J. Busch, Grigorij Schleifer, Dominik Schaack, Felix Lasitschka, Kenneth D. Bloch, Donald B. Bloch, Fumito Ichinose

**Affiliations:** 1 Anesthesia Center for Critical Care Research, Department of Anesthesia, Critical Care and Pain Medicine, Massachusetts General Hospital and Harvard Medical School, 55 Fruit Street, Boston, MA 02114 USA; 2 Department of Anesthesiology and Intensive Care Medicine, Campus Virchow-Klinikum, Charité Universitätsmedizin, Berlin, Germany; 3 Department of Anesthesiology, Ruprecht Karls University, Heidelberg, Germany; 4 Institute of Pathology, Ruprecht Karls University, Heidelberg, Germany; 5 Division of Rheumatology, Allergy and Immunology, Department of Medicine, Massachusetts General Hospital and Harvard Medical School, Boston, USA

**Keywords:** Hypoxic pulmonary vasoconstriction, Endotoxemia, Arginase, Nitric oxide synthase

## Abstract

**Background:**

Hypoxic pulmonary vasoconstriction (HPV) optimizes the match between ventilation and perfusion in the lung by reducing blood flow to poorly ventilated regions. Sepsis and endotoxemia impair HPV. We previously showed that nitric oxide synthase 2 (NOS2) is required, but not sufficient, for the effect of endotoxin on HPV. The aim of the current study was to identify additional factors that might contribute to the impairment of HPV during endotoxemia.

**Methods:**

Gene expression profiling was determined using pulmonary tissues from *NOS2-*deficient (*NOS2*^*−/−*^) and wild-type mice subjected to endotoxin or saline challenge (control). HPV was accessed as the percentage increase in left pulmonary vascular resistance (LPVR) in response to left main bronchus occlusion (LMBO) in wild-type mice.

**Results:**

Among the 22,690 genes analyzed, endotoxin induced a greater than three-fold increase in 59 and 154 genes in the lungs of wild-type and *NOS2*^*−/−*^ mice, respectively. Of all the genes induced by endotoxin in wild-type mice, arginase 1 (*Arg1*) showed the greatest increase (16.3-fold compared to saline treated wild-type mice). In contrast, endotoxin did not increase expression of *Arg1* in NOS2^−/−^ mice. There was no difference in the endotoxin-induced expression of *Arg2* between wild-type and NOS2-deficient mice. We investigated the role of arginase in HPV by treating the mice with normal saline or the arginase inhibitor N^ω^-hydroxy-nor-L-arginine (norNOHA). In control mice (in the absence of endotoxin) treated with normal saline, HPV was intact as determined by profound LMBO-induced increase in LPVR (121 ± 22% from baseline). During endotoxemia and treatment with normal saline, HPV was impaired compared to normal saline treated control mice (33 ± 9% vs. 121 ± 22%, *P* < 0.05). HPV was restored in endotoxin-exposed mice after treatment with the arginase inhibitor norNOHA as shown by the comparison to endotoxemic mice treated with normal saline (113 ± 29% vs, 33 ± 9%, *P* < 0.05) and to control mice treated with normal saline (113 ± 29% vs, 121 ± 22%, *P* = 0.97).

**Conclusions:**

The results of this study suggest that endotoxemia induces *Arg1* and that arginase contributes to the endotoxin-induced impairment of HPV in mice.

**Electronic supplementary material:**

The online version of this article (10.1186/s12931-019-1062-6) contains supplementary material, which is available to authorized users.

## Background

Hypoxic pulmonary vasoconstriction (HPV) diverts blood flow away from poorly- to better-ventilated regions of the lung, thereby optimizing gas exchange. The sensor and effector mechanisms responsible for HPV are intrinsic to pulmonary arterial smooth muscle cells [1]. Although it has been shown that HPV is modulated by a number of vasoactive mediators including nitric oxide (NO) and arachidonic acid metabolites, the precise mechanisms that mediate HPV remain incompletely understood [1–5].

HPV is impaired in patients with sepsis and in animal models of endotoxemia [2–7]. Impaired HPV (i.e., reduced pulmonary vasoconstrictor response in hypoxic lung regions) contributes to systemic hypoxemia during sepsis or endotoxemia. NO is produced by NO synthases (NOS1, NOS2, and NOS3). While NOS3 is constitutively expressed in vascular endothelial cells and importantly regulates pulmonary vascular tone, NOS2 is induced by inflammatory stimuli such as endotoxin. In a previous study, we showed that endotoxin impairs HPV in wild-type mice but not in mice with congenital *NOS2* deficiency (*NOS2*^*−/−*^) leading to lower arterial oxygen tensions in wild-type mice than in *NOS2*^*−/−*^ mice after endotoxin challenge. In saline-treated wild-type and *NOS2*^*−/−*^ mice, the inhalation of 40 ppm NO for 22 h did not affect HPV, suggesting that a high level of NO, by itself, is not sufficient to impair HPV. When endotoxemic wild-type and *NOS2*^*−/−*^ mice inhaled 40 ppm NO for 22 h, HPV was impaired to the same degree in both genotypes [2]. The results of these studies show that NO and at least one additional endotoxin-induced factor are required for endotoxin to impair HPV.

In the current study, we hypothesized that one or more gene(s) are differentially expressed in the lungs of wild-type and *NOS2*^−/−^ mice after endotoxin challenge. We used an unbiased transcriptional array approach to identify genes that may have increased or decreased expression after endotoxin exposure in wild-type mice, but not *NOS2*^−/−^ mice. Here, we report that expression of *arginase 1 (Arg1*) was markedly increased in the lungs of wild-type mice after endotoxin challenge. Endotoxin marginally decreased the expression of *Arg1* in *NOS2*^−/−^ mice. The arginase inhibitor N^ω^-hydroxy-nor-L-arginine restored HPV in wild-type mice. These observations identify a critical role for arginase in endotoxin-induced impairment of HPV.

## Materials and methods

### Animals and experimental setting

Male *NOS2*^*−/−*^ mice and their wild-type controls (C57BL/6 J) were purchased from The Jackson Laboratory (Bar Harbor, Maine). The body weight ranged between 21 and 27 g. Mice were challenged with intravenous administration of either normal saline (0.1 ml/10 g body weight) or lipopolysaccharide (LPS, *Escherichia coli* O111:B4, Sigma, St Louis, MO; 20 mg/kg, dissolved in normal saline 0.1 ml/10 g body weight).

### Microarray analysis

For microarray analysis, lungs were harvested from wild-type mice 22 h after challenge with either saline (*N* = 4) or LPS (*N* = 3) or from *NOS2*^*−/−*^ mice 22 h after saline (*N* = 1) or LPS (*N* = 3) challenge. RNA, cDNA, and labeled cRNA were generated as previously described [8]. Fragmented cRNA was hybridized to Affymetrix mouse genome MOE430A Array containing 22,690 gene entries with the GeneChip Fluidics Station 450 and scanned with the GeneChip® Scanner 3000. Affymetrix GeneChip 5.0 software was used for data analysis according to Affymetrix protocols. Microarray data processing was performed in the R environment. Bioconductor package “affy” was used for data input, background correction, normalization and calculation of expression values. Data sets were annotated with official gene symbols originating from package “moe430a.db” using “annotate”. Resulting gene sets were annotated using in-house scripts and technical information from Affymetrix.

### Measurement of gene expression

To confirm the increase in pulmonary *Arg1* expression found in the microarray analysis of endotoxemic wild-type mice, but not *NOS2*^*−/−*^ mice, additional wild-type and *NOS2*^*−/−*^ mice received either normal saline or LPS (*N* = 6 for each of the four groups). Twenty-two hours later, total RNA was isolated from homogenized lungs using Trizol (Invitrogen Life Technologies, Carlsbad, CA, USA). cDNA was generated with MMLV reverse transcriptase (Promega, Madison, WI, USA) and random primers (Promega, Madison, WI, USA). Real-time qPCR was performed using the ABI Prism 7000 Sequence Detection System (Applied Biosystems, Foster City, CA, USA) using FAM MGB primers for *Arg1* and *arginase 2 (Arg2*, Applied Biosystems, Foster City, CA, USA). 18S ribosomal RNA was detected using 18S VIC MGB primers (Applied Biosystems, Foster City, CA, USA) and Taqman Universal PCR Master Mix (Applied Biosystems, Foster City, CA, USA). Changes in *Arg1* and *Arg2* mRNA expression were determined using the ∆∆Ct method with normalization to 18S ribosomal RNA.

### Measurement of Arg1 protein expression

Immunoblots were performed to assess levels of Arg1 and GAPDH. Lungs from wild-type and *NOS2*^*−/−*^ mice with and without 22 h of endotoxemia were harvested, homogenized at 4 °C in PBS with 5 mM EGTA and protease inhibitor mix (Roche Diagnostics GmbH, Germany), and centrifuged at 10,000 g at 4 °C for 10 min. Protein in the supernatant protein was subjected to electrophoresis, transferred to a PVDF membrane, and probed with anti-Arg1 antiserum (1500, Lifespan Biosciences, Seattle, WA, USA) and anti-GAPDH antiserum (1:10,000, MerckMillipore, Darmstadt, Germany). Proteins were visualized using a LICOR infrared imager (Odyssey CLx) and quantitative densitometric analysis was performed (Odyssey Image Studio v3.1). The amount of arginase relative to GAPDH was determined.

### Immunoenzyme staining

To localize *Arg1* expression, lungs of wild-type mice were fixed in paraformaldehyde 22 h after challenge with saline (*N* = 5) or endotoxin (*N* = 5). Immunoenzyme stainings were performed on 2 μm paraffin-embedded sections using standard avidin-biotin anti-alkaline phosphatase technique (Vector Laboratories, Burlingame, CA) according to the manufacturer’s instructions. Tris-buffered saline supplemented with 0.2% bovine serum albumin (Biotrend, Cologne, Germany) was used as buffer. Primary antibody dilutions of rabbit polyclonal antibody, 1/50 (H-52, Santa Cruz, Dallas, Texas) and an isotype- and concentration-matched rabbit control Ig (Dianova, Hamburg, Germany) were prepared in this buffer and incubated for 1 h at room temperature. A biotinylated donkey anti-rabbit IgG Ab, 1/100 (Jackson ImmunoResearch, Newmarket, UK), was used as a secondary reagent (30 min at room temperature). Naphthol AS-biphosphate (Sigma) with New-fuchsin (Merck, Darmstadt, Germany) was used as the substrate for alkaline phosphatase. Slides were counterstained with hematoxylin (Sigma).

### Measurement of hypoxic pulmonary vasoconstriction in mice

To investigate the effect of arginase on HPV, mice were initially challenged with either LPS or normal saline (control). Twenty-one hours later, mice were treated with an intravenous bolus of the arginase inhibitor N^ω^-hydroxy-nor-L-arginine (norNOHA, Cayman Chemical, Ann Arbor, Michigan; 20 mg/kg or 40 mg/kg) or normal saline. One hour later, HPV was measured as described previously [3–5, 7]. Mice were anesthetized and mechanically ventilated with a respiratory frequency of 100–110 breaths per minute, a tidal volume of 10 ml/kg body weight, the peak inspiratory pressure of approximately 10 cm H_2_O, positive end-expiratory pressure 2 cm H_2_O, and at an inspired oxygen fraction of 1.0. An arterial line was placed in the left carotid artery, and a left-sided thoracotomy was performed. A custom-made polyethylene catheter was positioned in the main pulmonary artery, and a flow probe was placed around the left pulmonary artery. Heart rate, systemic arterial pressure (SAP), pulmonary arterial pressure (PAP), and left pulmonary arterial blood flow (QLPA) were continuously measured and recorded (DI 720; Dataq Instruments, Akron, OH). Left lung alveolar hypoxia and collapse was induced by left mainstem bronchus occlusion (LMBO). A 50% transient occlusion of the inferior vena cava was repeated three times before and during LMBO to estimate left pulmonary vascular resistance (LPVR). LPVR was calculated from the slope of the PAP/QPLA relationship. The LMBO-induced increase in LPVR was expressed as the percentage increase of LPVR during LMBO from the baseline.

### Measurement of arterial blood gases

After hemodynamic measurements were obtained, blood was sampled from the left carotid artery, anticoagulated with heparin, and blood gas analyses were performed using a Rapid Lab 840 (Chiron Diagnostics, Medfield, MA).

## Statistical analysis

Data are expressed as mean ± SD. *P* values less than 0.05 were considered statistically significant. Statistical analyses were performed using Sigma Stat 3.0 (Systat Software Inc., Richmond, CA). Normality distribution of data was checked using the Kolmogorov-Smirnov test with Lillefors correction. For the comparison between the groups, data were analyzed using ANOVA with a post-hoc Tukey test for normally distributed data or using Kruskal Wallis ANOVA on ranks with a post-hoc Turkey test for not normally distributed data. Hemodynamic changes before (baseline) and during LMBO were compared with a paired t-test.

For the microarray data, the differential expression gene set annotation was performed as follows. The data analysis from wild-type and *NOS2*^*−/−*^ mice after challenge with either saline or LPS was conducted with “limma” (Linear Models for Microarray and RNA-Seq, https://bioconductor.org) [9]. Empirical Bayes statistics for differential expression was calculated for both genotypes and conditions. To allow comparison of data sets moderated F-statistics were applied. Differentially up-regulated genes were selected that showed linear expression values of at least 3-fold after 22 h after LPS challenge in comparison to saline challenge. Gene candidates were considered as down-regulated if the respective expression values were decreased to 0.3-fold or less. Probability values were adjusted for multiple testing using the Benjamini–Hochberg procedure. Genes that were either up-regulated (≥ 3.0) or down-regulated (≤ 0.3) after treatment with LPS compared to saline were identified in *NOS2*^*−/−*^ and wild-type mice. Then genes with endotoxin-induced increased expression (≥ 3.0) or decreased expression (≤ 0.3) were compared in wild-type and *NOS2*^*−/−*^ mice. The Supplemental files summarize the results as follows: Additional file 1. Genes with increased expression in endotoxemic wild-type, but not in endotoxemic *NOS2*^*−/−*^ mice; Additional file 2. Genes with decreased expression in endotoxemic wild-type, but not endotoxemic *NOS2*^*−/−*^ mice; Additional file 3. Genes with increased expression in endotoxemic *NOS2*^*−/−*^, but not endotoxemic wild-type mice; Additional file 4. Genes with a decreased expression endotoxemic *NOS2*^*−/−*^, but not endotoxemic wild-type mice.

## Results

### Endotoxin challenge increases *Arg1* levels in the lungs; dependence on NOS2

**Fig. 1:**
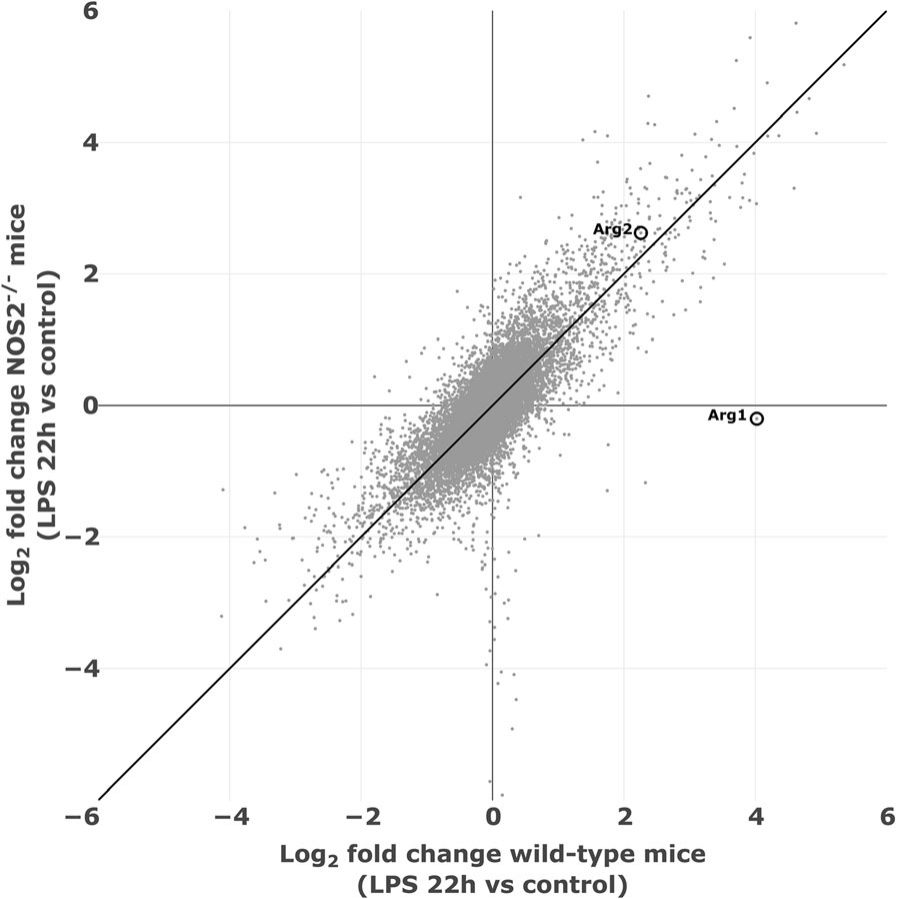
Up-regulated genes in the lungs of endotoxemic wild-type, but not NOS2-deficient mice. Whole lung tissues from wild-type mice treated with (*N* = 3), and without (*N* = 4), endotoxin and from *NOS2*^*−/*−^ mice treated with (*N* = 3), and without (*N* = 1), LPS were studied*.* The grey dots show the log2 fold change in gene expression upon LPS exposure; the x-axis represents changes in wild-type mice and the y-axis changes in *NOS2*^*−/−*^ mice. The transcriptional profiles revealed a contrasting gene expression of pulmonary *Arg1* in wild-type and *NOS2*^*−/−*^ mice after LPS challenge: *Arg1* expression increased in endotoxemic wild-type mice compared to saline challenged wild-type mice (positive scaling on the x-axis). In contrast, *Arg1* expression did not increase in endotoxemic *NOS2*^*−/−*^ mice compared to the saline-treated *NOS2*^*−/−*^ mice (negative scaling on the y-axis). *Arg2* expression increased in both strains after LPS challenge (positive scaling on the x- and y-axis).

Endotoxin impairs HPV in wild-type mice, but not in *NOS2*^*−/−*^ mice [2]. We hypothesized that differences in endotoxin-induced gene expression between wild-type mice and *NOS2*^*−/−*^ mice might explain the different response to endotoxin in the two genotypes. Therefore, we compared the transcriptional profile of lungs from wild-type and *NOS2*^*−/−*^ mice 22 h after endotoxin challenge using RNA microarrays and analyzed the data for up- and down-regulated gene expression. The microarrays assessed the expression of 22,690 genes. Compared to the lungs of control wild-type mice treated with saline, endotoxin challenge induced a greater than three-fold increase in 59 genes and a three-fold decrease in 72 genes in the lungs of wild-type mice (Additional file 1 and Additional file 2). Compared to the lungs of *NOS2*^*−/−*^ mice challenged with saline, LPS caused an up-regulation of 154 genes and a down-regulation of 93 genes in the lungs of *NOS2*^*−/−*^ mice (Additional file 3 and Additional file 4). Of the 800 genes that had three-fold altered expression by endotoxin, 346 were up-regulated or down-regulated in the lungs of both genotypes (Fig. 1). However, the results of the microarray analysis indicated that the effect of endotoxin on *Arg1* expression was discordant in the two genotypes: Expression of *Arg1* was up-regulated 16.3-fold in the lungs of LPS-treated wild-type mice; LPS induced a 10% decrease in the expression of *Arg1* in LPS-treated *NOS2*^*−/−*^ mice. In wild-type mice, LPS challenge induced a 4.8-fold increase in *Arg2* gene expression compared to saline-treated wild-type mice. Exposure of *NOS2*^*−/−*^ mice to LPS caused a similar, 6.1-fold increase in *Arg2* gene expression compared to saline challenged *NOS2*^*−/−*^ mice. These results suggest that endotoxin up-regulates *Arg1* expression in the lungs of wild-type, but not *NOS2*^*−/−*^ mice, while endotoxemia induces *Arg2* expression to a similar extent in both genotypes.

**Fig. 2:**
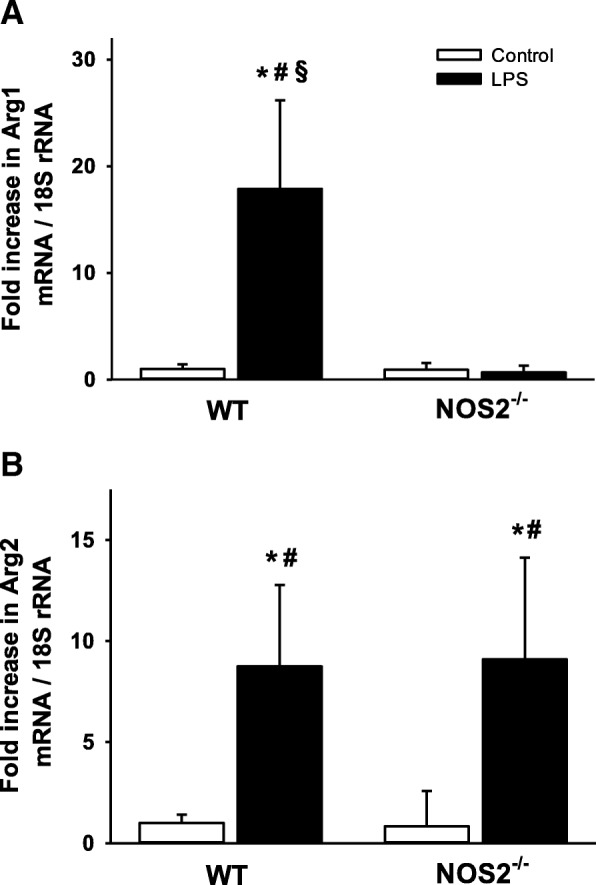
Pulmonary mRNA expression of *Arg1* and *Arg2* in wild-type mice and *NOS2*^*−/−*^ mice. Endotoxin increased the pulmonary *Arg1* mRNA expression in wild-type mice but not in *NOS2*^*−/−*^ mice (Panel **a**). The pulmonary *Arg2* mRNA expression was similar in wild-type mice and *NOS2*^*−/−*^ mice after LPS challenge (Panel **b**). Tissue samples were taken 22 h after challenge with either saline (control) or LPS. *N* = 6 per group; data are presented as mean ± SD; * *P* < 0.05 vs. saline-challenged wild-type mice, # *P* < 0.05 vs. saline-challenged *NOS2*^*−/−*^ mice, and § *P* < 0.05 vs. endotoxin-challenged *NOS2*^*−/−*^ mice

**Fig. 3:**
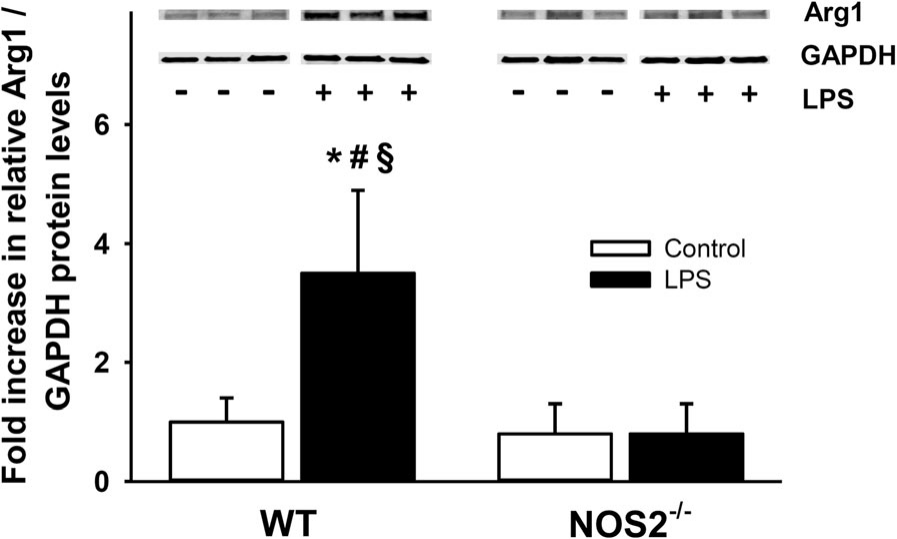
Pulmonary expression of *Arg1* in wild-type and NOS2^−/−^ mice. The level of *Arg1* protein was low in the lungs of saline-treated wild-type mice (*N* = 6) and was markedly increased in the lungs of wild-type mice treated with LPS (*N* = 6). *Arg1* protein level were low in NOS2^−/−^ mice challenged with saline (control, *N* = 4) or endotoxin (*N* = 5). Lungs were harvested 22 h after challenge with either saline (control) or lipopolysaccharide (LPS). Data are presented as mean ± SD; * *P* < 0.05 vs. saline-challenged wild-type mice, # *P* < 0.05 vs. saline-challenged *NOS2*^*−/−*^ mice, and § *P* < 0.05 vs. endotoxin-challenged *NOS2*^*−/−*^ mice

To verify the microarray results, pulmonary mRNA levels of *Arg1* and *Arg2* were measured using qPCR. LPS challenge increased *Arg1* mRNA levels in the lungs of wild-type mice (17.9-fold increase compared to saline-challenged wild-type mice, *P* < 0.05) but not in *NOS2*^*−/−*^ mice (0.67-fold decrease compared to saline challenged wild-type mice, *P* = 0.07) (Fig. 2a). Endotoxin increased *Arg2* mRNA levels similarly in the lungs of wild-type mice and *NOS2*^*−/−*^ mice (8.8-fold increase vs 9.1-fold increase, *P* = 0.90, Fig. 2b). Immunoblots were used to measure the protein levels of *Arg1* in the lungs of wild-type and *NOS2*^*−/−*^ mice after challenge with saline or LPS. LPS challenge increased the protein level of *Arg1* in wild-type, but not in *NOS2*^*−/−*^, mice (Fig. 3). Taken together, the results confirm that endotoxin challenge induces a marked increase in *Arg1* expression in the lungs. In contrast, induction of *Arg2* expression is similar in the two genotypes.

### Endotoxin increased *Arg1* immunoreactive protein expression in small pulmonary arteries, bronchial epithelium and connective tissue

**Fig. 4:**
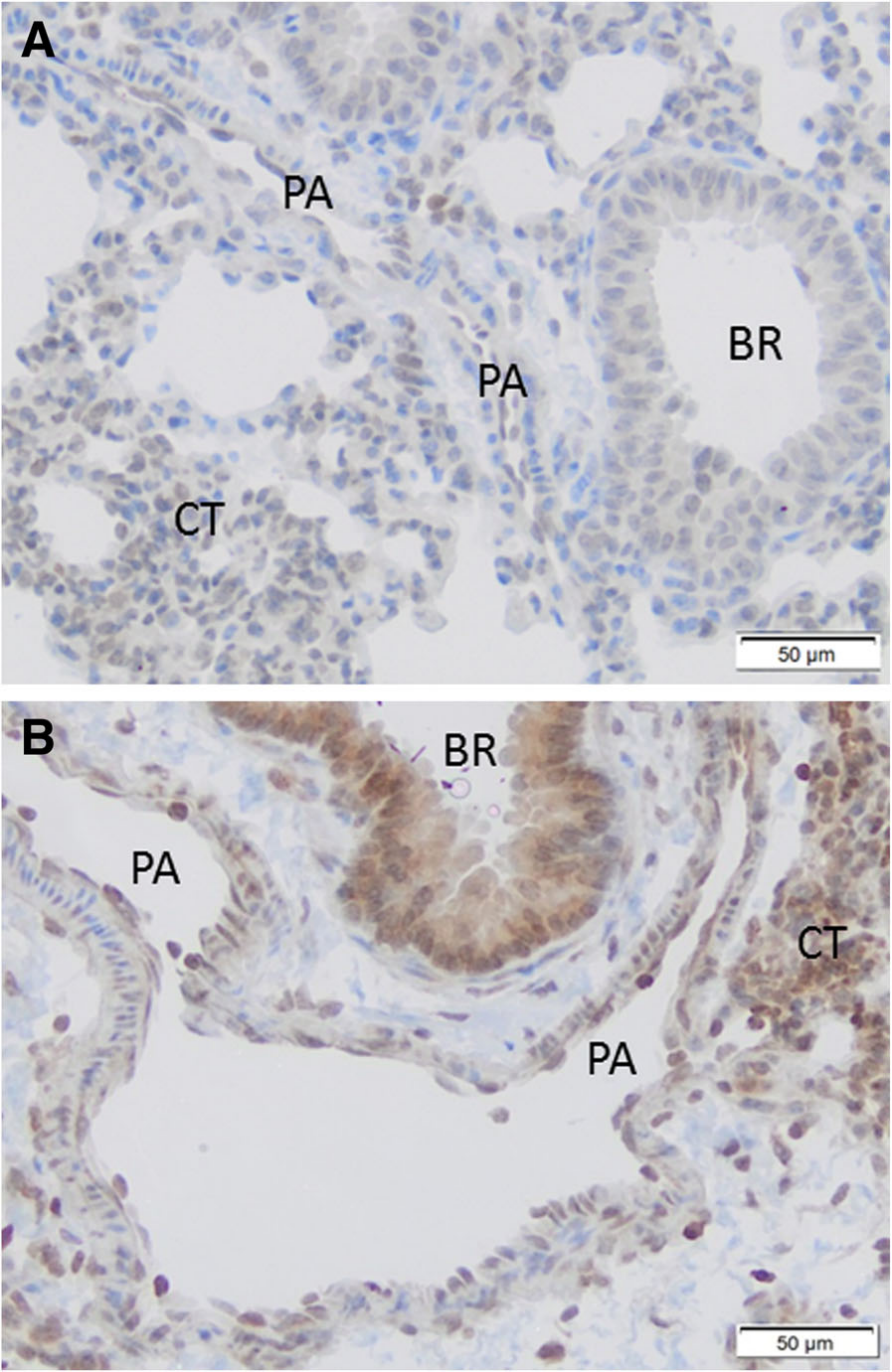
*Arg1* immunostaining in lung sections of wild-type mice. *Arg1* immunoreactivity in lungs of mice after saline (**a**) or endotoxin challenge (**b**). Immunoenzyme stainings were performed on paraffin-embedded sections using polyclonal rabbit anti-Arg1 and counterstained with hematoxylin. Representative image shows *Arg1* immunoreactive protein (purple-brown) in bronchial epithelium (BR), connective tissue (CT) and small pulmonary arteries (PA)

To evaluate the location of *Arg1* protein expression, lung sections of wild-type mice were stained with *Arg1* specific antibodies 22 h after challenge with saline (A) or endotoxin (B) (Fig. 4). *Arg1* expression was found in walls of small pulmonary arteries, bronchial epithelium, and in connective tissue.

### An arginase inhibitor, norNOHA, restores HPV after LPS challenge

To determine the physiological impact of arginase on HPV, we examined the effects of the arginase inhibitor norNOHA on HPV in anesthetized wild-type mice. At baseline, the hemodynamic parameters did not differ between the mice in five experimental groups: 1) saline-challenged control mice treated with normal saline 21 h after the initial challenge; 2) control mice treated with norNOHA 21 h after the saline challenge; 3) LPS-challenged mice treated with normal saline 21 h after the initial challenge; 4) LPS-challenged mice treated with 20 mg/kg norNOHA 21 h after the initial challenge, and 5) LPS-challenged mice treated with 40 mg/kg norNOHA 21 h after the initial challenge (Table 1).

**Table 1:** Hemodynamic measurements

	Control		LPS		
	NS	norNOHA (40mg/kg)	NS	norNOHA (20mg/kg)	norNOHA (40mg/kg)
HR (bpm)					
Baseline	593±50	619±57	605±61	651±54	631±62
LMBO	584±44	626±71	603±50	634±74	636±68
SAP (mmHg)					
Baseline	93±18	97±11	96±14	95±7	102±4
LMBO	91±12	77±7^A^*	93±16*	92±9	101±6
PAP (mmHg)					
Baseline	17±2	17±1	18±2	16±2	17±1
LMBO	19±1*	19±1*	20±1*	19±4*	20±2*
QLPA (ml/min)					
Baseline	2.3±0.3	2.6±0.5	2.8±0.4	2.9±0.9	2.4±0.3
LMBO	1.4±0.2*	1.9±0.5*	2.3±0.4^B^*	2.6±1.0^AB^	1.6±0.3*

Hemodynamic parameters before (baseline) and during left mainstem bronchus occlusion (LMBO) in wild-type mice 22 h after challenge with saline (control) or endotoxin (LPS). One hour prior to the measurement, mice were treated with normal saline (NS) or with the arginase inhibitor Nω-hydroxy-nor-Arginine (norNOHA). N = 7 per group

*HR* heart rate, *SAP* systemic arterial pressure, *PAP* pulmonary arterial pressure, *QLPA* flow rate in the left pulmonary artery, *bpm* beats per minute, *mmHg* millimeters of mercury, and ml/min milliliter per minute

Data are presented as mean ± SD. A P < 0.05 vs. LPS norNOHA 40 mg/kg, B P < 0.05 vs. control NS, * P < 0.05 vs. baseline

During LMBO, the heart rates were comparable between the groups. The SAP remained stable before and during LMBO in all groups except control mice that were treated with norNOHA. In this group, the SAP decreased compared to baseline (97 ± 11 mmHg vs. 77 ± 7 mmHg, *P* < 0.05), suggesting that the arginase inhibitor caused systemic vasodilation. Endotoxemic mice that received normal saline (instead of norNOHA) had a small but statistically significant decrease in SAP during LMBO compared to baseline (96 ± 14 mmHg vs. 93 ± 16 mmHg**,** *P* < 0.05). The PAP increased compared to baseline during LMBO in each group (Table 1).

**Fig. 5:**
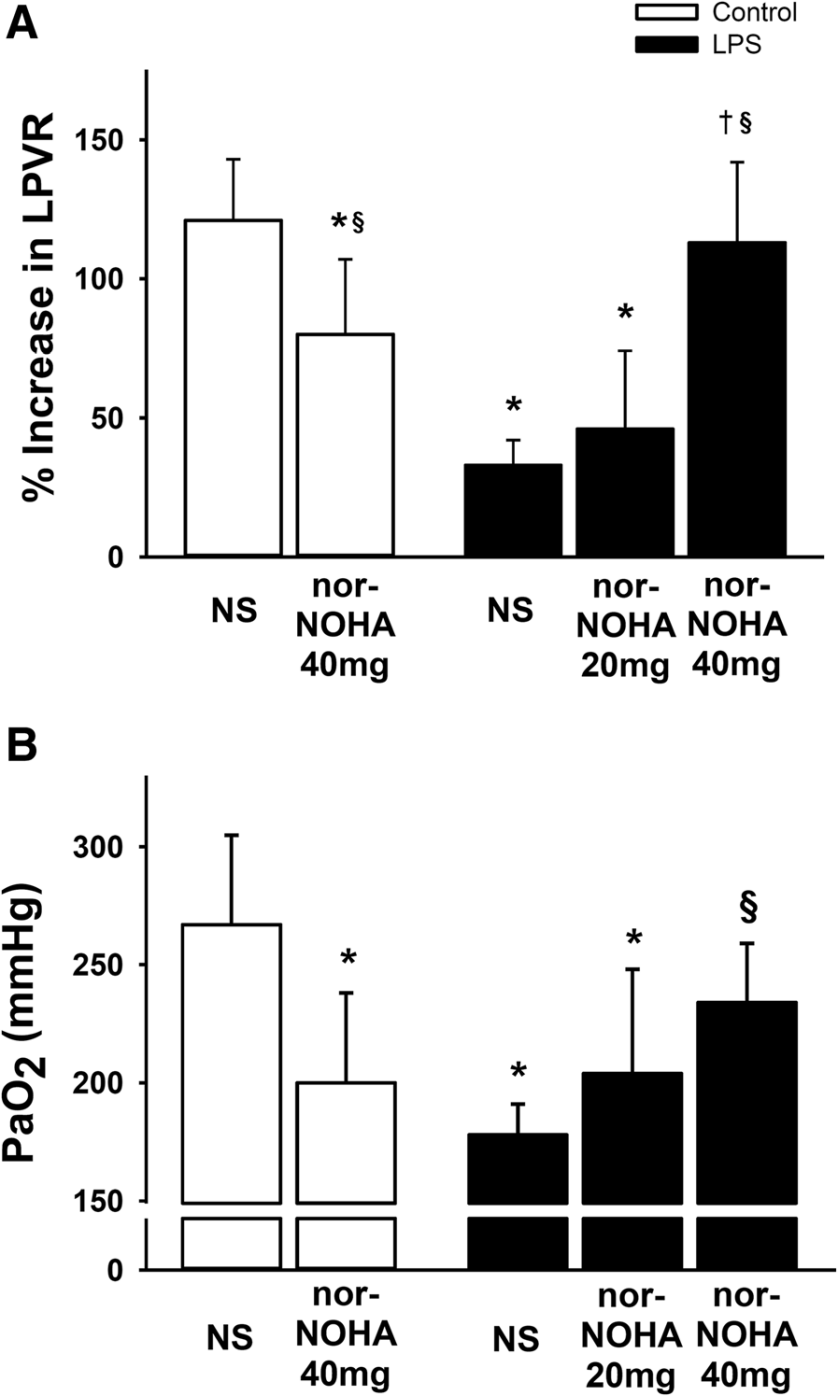
Hypoxic pulmonary vasoconstriction and arterial oxygenation. Panel A: Mice were challenged with saline (control) or endotoxin (LPS). After 21 h, the animals were treated with normal saline (NS) or the arginase inhibitor norNOHA (20 mg/kg or 40 mg/kg). HPV was assessed as the left pulmonary vascular resistance (LPVR) in response to left mainstem bronchus occlusion (LMBO). Treatment with the arginase inhibitor norNOHA (40 mg/kg) restored HPV in endotoxemic mice. Panel B: Arterial blood gas tensions were measured during LMBO at the end of the experiments. The restoration of HPV was associated with improved arterial oxygenation (PaO_2_) in endotoxemic mice treated with 40 mg/kg norNOHA compared to saline treatment. *N* = 7 per group; data presented as mean ± SD; * *P* < 0.05 vs. control group treated with NS, § *P* < 0.05 vs. LPS group treated with normal saline, † LPS group treated with 20 mg/kg norNOHA

To investigate the effect of arginase inhibition on HPV, we measured the percentage increase in LPVR before and during LMBO. In control mice treated with normal saline, LMBO caused a ~ 121% increase in LPVR from baseline (Fig. 5a). In control mice treated with norNOHA, the LPVR response to LMBO was attenuated compared to control mice treated with saline (80 ± 27% vs 121 ± 22%, *P* < 0.05). Mice challenged with LPS and treated with normal saline had an impaired increase in LPVR in response to LMBO compared to control mice treated with normal saline (33 ± 9% vs. 121 ± 22%, *P* < 0.05). The increase in LPVR in response to LMBO was also smaller in the LPS-challenged mice treated with norNOHA at 20 mg/kg compared to the control mice treated with normal saline (46 ± 28% vs. 121 ± 22%, *P* < 0.05). In contrast, treatment with norNOHA at 40 mg/kg restored the increase in LPVR in response to LMBO in LPS-challenged mice to a level that was comparable to that of control mice treated with normal saline (113 ± 29% vs 121 ± 22%, *P* = 0.97, Fig. 5a). These results show that the arginase inhibitor norNOHA, at a dosage of 40 mg/kg but not 20 mg/kg, restores HPV when administered to mice 22 h after endotoxin challenge.

### Restoration of HPV in endotoxemic mice improves systemic arterial oxygen tensions during LMBO

To estimate the impact of HPV on intrapulmonary shunt and systemic oxygenation, arterial blood gas tensions were measured during LMBO at the end of the HPV measurements (Fig. 5a, Table 2). The arterial pH of endotoxemic mice treated with normal saline, 20 or 40 mg/kg of norHOHA was lower than the arterial pH of control mice treated with normal saline (*P* < 0.05 for all three comparisons) or control mice treated with 40 mg/kg norNOHA (*P* < 0.05 for all three comparisons). Accordingly, the arterial base excess of endotoxemic mice after treatment with normal saline, 20 or 40 mg/kg norNOHA was smaller than the arterial base excess of control mice that received normal saline (*P* < 0.05 for all three comparisons) or control mice treated with 40 mg/kg norNOHA (*P* < 0.05 for all three comparisons). There was no difference in the arterial carbon dioxide tension between all experimental groups, suggesting that endotoxin induced a metabolic acidosis. The hemoglobin concentrations were similar in all mice. The arterial oxygen tension (P_a_O_2_) was highest in control mice treated with normal saline during LMBO. There was a small but significant decrease in P_a_O_2_ in control mice treated with norNOHA compared to normal saline (200 ± 38 mmHg vs. 267 ± 38 mmHg, *P* < 0.05). During LMBO, the P_a_O_2_ was lower in LPS-challenged mice treated with normal saline or 20 mg/kg norNOHA compared to control mice treated with normal saline (*P* < 0.05 for each comparison). In contrast, treatment of endotoxemic mice with 40 mg/kg norNOHA restored the P_a_O_2_ to levels that were comparable to those in control mice (not challenged with LPS) treated with saline (not treated with norNOHA) (234 ± 25 mmHg vs. 267 ± 38 mmHg, *P* = 0.35). These results suggest that treatment with 40 mg/kg norNOHA restores HPV and decreases intrapulmonary shunt in endotoxemic mice with an improvement in systemic arterial oxygenation.

**Table 1:** Arterial blood gas analyses

	Control		LPS		
	NS	norNOHA (40mg/kg)	NS	norNOHA (20mg/kg)	norNOHA (40mg/kg)
P_a_O_2_ (mmHg)	267±38	200±38*	178±13*	204±44*	234±25^§^
P_a_CO_2_ (mmHg)	30.9±5.1	30.9±3.5	34.0±5.0	36.7±8.4	30.7±4.8
pH_a_ (mmHg)	7.31±0.10	7.37±0.05	7.13±0.07*‡	7.07±0.08*‡	7.11±0.04*‡
BE (mmol/l)	-9.0±5.1	-6.4±2.0*	-17.5±1.6*‡	-18.4±4.6*‡	-19.7±2.5*‡
Hb (g/dl)	13.0±1.0	13.0±1.1	11.8±1.1	11.7±1.4	11.8±1.3

Mice were challenged with saline (control) or endotoxin (LPS). After 21 h, animals received treatment with normal saline (NS) or the arginase inhibitor Nω-hydroxy nor-Arginine (norNOHA). Blood gas analyses were performed during occlusion of the left mainstem bronchus. N = 7 per group

*P*_*a*_*O*_*2*_ arterial oxygen tension, *P*_*a*_*CO*_*2*_ arterial carbon dioxide tension, *pH*_*a*_ arterial pH, *BE* base excess, *Hb* hemoglobin, *mmHg* millimeters of mercury, *mmol / l* milimoles per liter, *g/dl* gram per deciliter

Data are presented as mean ± SD. * P < 0.05 vs. control NS, § P < 0.05 vs. LPS NS, ‡ P < 0.05 vs. control norNOHA

## Discussion

The current study used expression microarrays to identify one or more genes that might contribute, together with NO, to the endotoxin-induced impairment of HPV. Transcriptional profiling revealed that *Arg1* was the most up-regulated gene in the lungs of wild-type mice after endotoxin challenge and that *Arg1* was marginally decreased in endotoxemic *NOS2*^*−/−*^ mice. The endotoxin-induced increase in *Arg1* mRNA in wild-type, but not in *NOS2*^*−/−*^ mice was confirmed using qPCR. Immunoblots showed that the differences in Arg1 mRNA levels resulted in corresponding differences in Arg1 protein levels. Immunohistochemical analysis revealed that endotoxin induced *Arg1* expression in the walls of small pulmonary artery, bronchial epithelium, and connective tissues in wild-type mice. The functional importance of arginase in the endotoxin-induced impairment of HPV was supported by the restoration of HPV by the arginase inhibitor norNOHA. Taken together, these results reveal a critical role for arginase in the impairment of HPV during endotoxemia in mice.

**Fig. 6:**
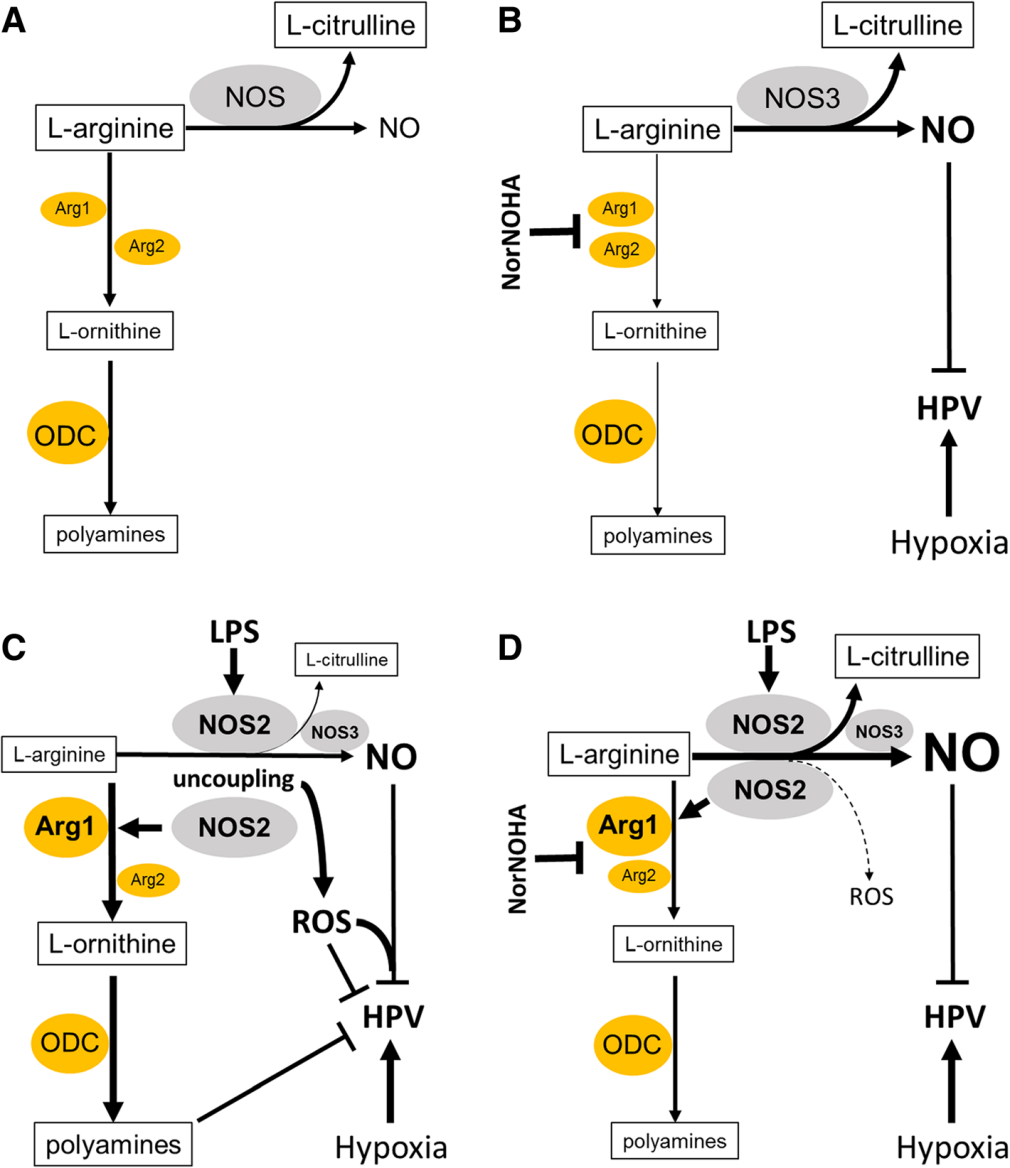
Hypothetical roles of arginase and arginase inhibition on HPV. **a** Arginase and nitric oxide synthase (NOS) compete for the common substrate L-arginine. **b** In healthy control mice, the arginase inhibitor norNOHA may shift L-arginine to NOS thereby increasing the level of NO that in turn attenuates HPV. **c** Endotoxemia increases expression of NOS2 and *Arg1*. NOS2-dependent S-nitrosylation may promote *Arg1* expression and activity. Increased *Arg1* may decrease L-arginine concentration locally. Low local arginine levels might promote NOS2 uncoupling and reactive oxygen species (ROS) production. Increased NO levels and redox imbalance impairs HPV. Upregulated *Arg1* also potentially increases downstream products including L-ornithine and polyamines, which may also impair HPV. **d** Inhibition of arginase by norNOHA restores HPV by preventing NOS2 uncoupling and ROS production by shifting more L-arginine to NOS2 and decreasing polyamines

The production of NO by NOS depends on the availability of L-arginine because no other amino acid or guanidino-containing compound can substitute as a substrate for NOS. L-arginine is metabolized by NOS to NO and L-citrulline and by arginase to urea and L-ornithine (Fig. 6a) [10]. Because L-arginine is the substrate for both NOS and arginase, one might expect that inhibition of arginase would increase the availability of L-arginine for NOS to produce more NO and thereby impair HPV. In this study, the pulmonary level of *Arg1* and *Arg2*, were low at baseline (Figs. 2 and 3), suggesting that inhibition of arginase would have little effect on healthy mice. However, we observed that administration of norNOHA to healthy control mice modestly attenuated HPV. It is possible that, even in the presence of low baseline levels of arginase, norNOHA was able to increase the level of L-arginine available to NOS (primarily NOS3), resulting in more NO production, which exerted pulmonary vasodilating effects that oppose HPV (Fig. 6b).

To the best of our knowledge, this is the first study that showed that NOS2 is required for the ability of endotoxin to induce *Arg1* in the lung. Although detailed signaling mechanism of NOS2-dependent *Arg1* induction was not explored in the current study, protein S-nitrosylation induced by NOS2-derived NO has been shown to regulate transcription. For example, NOS2-dependent S-nitrosylation inhibits deacetylase sirtuin1 (SIRT1) during inflammation [11]. Inhibition of SIRT1 in turn activates signal transducer and activator of transcription 3 (STAT3) by increasing acetylation and phosphorylation of STAT3 [12]. Therefore, NOS2-dependent S-nitrosylation may activate STAT3-dependent transcription. Because STAT6/STAT3 are known promoters of *Arg1* expression, it is conceivable that endotoxin-induced NOS2 upregulated *Arg1* expression via STAT3 activation [13].

In this study, endotoxin challenge increased *Arg1* mRNA and protein expression in homogenized lung tissue. The immunohistochemistry revealed a marked increase of *Arg1* expression in the small pulmonary arteries, bronchial epithelium, and connective tissues. HPV is intrinsic to the pulmonary arterial smooth muscle cells. However, HPV is also modified by extrinsic factors derived from neighboring cell types such as vascular endothelial cells and macrophages [1]. *Arg1* is typically expressed in liver, red blood cells, and macrophages, but can be expressed in vascular endothelial cells and smooth muscle cells [14, 15]. It is conceivable that *Arg1* induction in multiple cell types modulates HPV during endotoxemia.

By reducing blood flow to poorly ventilated regions, HPV optimizes the match between ventilation and perfusion in the hypoxic regions of the lung and improves arterial oxygenation [1]. In this study, we observed that endotoxin challenge impaired HPV and decreased arterial oxygenation. In contrast, administration of arginase inhibitor during endotoxemia restored HPV and improved arterial oxygenation to the levels comparable to healthy control mice treated with normal saline. These results indicate Arg1 as a potential new therapeutic target for endotoxin-induced ventilation perfusion mismatch associated with systemic hypoxemia.

Twenty-two hours after endotoxin challenge, HPV was impaired in mice treated with normal saline, whereas administration of 40 mg/kg norNOHA restored HPV. Because inhibition of arginase can theoretically make more L-arginine available for NOS to produce NO, the observed protective effect of norNOHA on HPV is counterintuitive. However, the L-arginine levels, and therefore the substrate availability for NOS to produce NO, differs under healthy and pro-inflammatory conditions. Sepsis and endotoxemia reduce L-arginine levels [16, 17]. It has also been shown that NOS2-dependent S-nitrosylation activates *Arg1* by stabilizing *Arg1* trimer thus shifting more L-arginine to *Arg1*-dependent signaling [18]. In any case, decreased L-arginine availability can result in uncoupling of NOS (predominantly NOS2 under pro-inflammatory condition), with decreased production of NO and increased production of reactive oxygen species (ROS) (Fig. 6c) [19]. Increased oxidative stress and decreased anti-oxidant capacity have been reported in LPS-induced lung injury [19, 20]. We previously showed that treatment with ROS-scavengers attenuates endotoxin-induced impairment of HPV [7]. It has been reported that arginase inhibition preserves NOS coupling thereby reduces ROS production and vascular endothelial dysfunction in aging rats [21]. It is conceivable that norNOHA attenuated NOS-uncoupling, ROS production, and redox imbalance to preserve HPV in endotoxemic mice (Fig. 6d).

An alternative explanation for the observed restoration of HPV in endotoxemic mice treated with norNOHA is that the inhibitor may decrease the level of arginase downstream products, such as ornithine and polyamines. The polyamines putrescine, spermidine, and spermine are small polycationic molecules that rectify ion channel conductance [22]. Altered ion conductance in pulmonary artery smooth muscle cells modulates HPV [1]. Polyamine catabolism has also been shown to increase oxidative stress which may promote redox imbalance [23]. The potential role of arginine metabolites in certain cell populations (such as vascular smooth muscle cells) in the regulation of HPV remains to be defined.

It is possible that other factors, in addition to NOS2 and *Arg1*, contribute to the impairment of HPV. For example, endothelial protein C receptor (also known as activated protein C receptor) was the second most up-regulated gene in the lungs of endotoxemic wild-type mice after *Arg1* (Additional file 1). Richard and colleagues reported that administration of activated protein C worsened oxygenation, presumably due to increased ventilation-perfusion mismatching, in a porcine model of acute lung injury [24]. The potential role of the endothelial protein C receptor in regulating HPV during endotoxemia warrants further studies.

A potential limitation of this investigation is the small number of lungs that were used in the microarray analyses. However, the transcriptional profiling was designed as a pilot study to identify candidate genes. Additional qPCRs and immunoblots were performed to confirm that endotoxin induced *Arg1* gene expression in wild-type, but not *NOS2*^*−/−*^ mice. A second limitation concerns the immunohistochemistry. After endotoxin challenge, *Arg1* positive immunostaining was found in bronchial epithelium, small pulmonary arteries, and the connective tissue. However, no double staining was performed leaving the specific cell type uncertain. Further studies are needed to identify which cell types in lung tissue are responsible for increased arginase activity.

## Conclusions

In summary, transcriptional profiling identified *Arg1* as a factor that might be required for endotoxin to inhibit HPV in wild-type mice. The arginase inhibitor norNOHA, at a dose of 40 mg/kg, acutely restored HPV in endotoxemic mice. These results identify arginase as a novel molecular target to improve pulmonary ventilation-perfusion matching during endotoxemia.

## Additional files

Additional file 1: Up-regulated genes in the lungs of endotoxemic wild-type, but not NOS2-deficient mice. We previously showed that HPV is preserved in endotoxemic NOS2-deficient (*NOS2*^*−/−*^) mice. [2]. The objective of this study was to identify gene products that may contribute to endotoxin-induced impairment of HPV in wild-type mice. Transcription profiling was performed on homogenized lung tissue from wild-type mice 22 h after LPS administration (*N* = 3) and compared to the profile produced by the lungs of wild-type mice challenged with saline LPS (*N* = 4). Transcription profiling was also performed on homogenized lung tissue from *NOS2*^*−/−*^ mice 22 h after LPS administration (*N* = 3) and was compared to the profile produced by the lungs of *NOS2*^*−/−*^ mice challenged with saline (*N* = 1). The microarray assessed the expression of 22,690 genes. A subset of 59 genes (shown in this table) was increased greater than three-fold in wild-type mice challenged with endotoxin, but was not enhanced three-fold in the lungs of *NOS2*^*−/−*^ mice challenged with endotoxin. The microarray results indicated that *Arg1* was the most highly expressed gene in endotoxemic wild-type mice, and that *Arg1* expression was not increased in the lungs of endotoxemic *NOS2*^*−/−*^ mice. (DOCX 1424 kb)

Additional file 2: Down-regulated genes in the lungs of endotoxemic wild-type mice, but not endotoxemic NOS2-deficient mice. Expression of 72 genes was decreased to 0.3-fold or less by endotoxin in wild-type mice, but was not decreased by endotoxin in *NOS2*^*−/−*^ mice. (DOCX 2071 kb)

Additional file 3: Up-regulated genes in the lungs of endotoxemic *NOS2*^*−/−*^ mice, but not in endotoxemic wild-type mice. Expression of 155 genes was increased more than three-fold by endotoxin in *NOS2*^*−/−*^ mice, but was not similarly increased by endotoxin in wild-type mice. (DOCX 3039 kb)

Additional file 4: Down-regulated genes in the lungs of endotoxemic *NOS2*^*−/−*^ mice, but not in endotoxemic wild-type mice. Ninety-three genes were decreased to 0.3-fold or less by endotoxin in *NOS2*^*−/−*^ mice, but were not decreased by endotoxin in wild-type mice. (DOCX 2346 kb)

## Abbreviations

Arg1: Arginase 1
Arg2: Arginase 2
BE: Base excess
Hb: Hemoglobin
HPV: Hypoxic pulmonary vasoconstriction
HR: Heart rate
LMBO: Left main bronchus occlusion
LPS: Lipopolysaccharide
LPVR: Left pulmonary vascular resistance
NO: Nitric oxide
norNOHA: N^ω^-hydroxy-nor-L-arginine
NOS2: Nitric oxide synthase 2
NOS2^−/−^: NOS2-deficiency
NOS3: Nitric oxide synthase 3
NS: Normal saline
ODC: L-ornithine decarboxylase
PaCO_2_: Arterial carbon dioxide tension
PaO_2_: Arterial oxygen tension
PAP: Pulmonary arterial pressure
pH_a_: Arterial pH
Procr: Endothelial protein C receptor (activated protein C receptor)
QLPA: Left pulmonary arterial blood flow
ROS: Reactive oxygen species
SAP: Systemic arterial pressure
SIRT1: Sirtuin1
STAT3: Signal transducer and activator of transcription 3
