# Supplementary figures and images for: Arginase impairs hypoxic pulmonary vasoconstriction in murine endotoxemia
Source: Respir Res. 2019 Jun 3;20:109. doi: 10.1186/s12931-019-1062-6 (PMC6547543; doi:10.1186/s12931-019-1062-6)

**Additional file 2: Table S2**


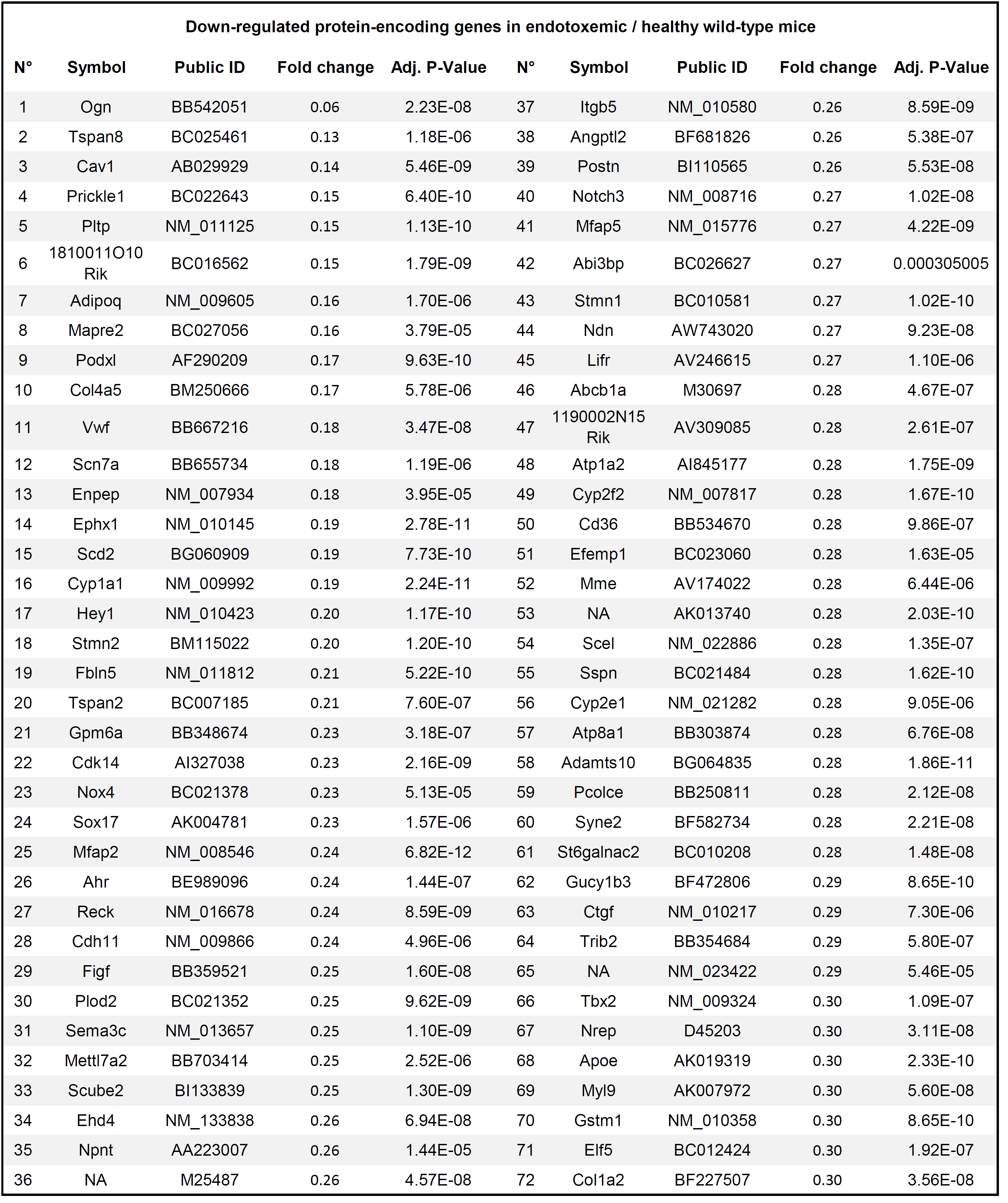

Supplement: Supplementary file 2 — Down-regulated genes in the lungs of endotoxemic wild-type mice, but not endotoxemic NOS2-deficient mice. Expression of 72 genes was decreased to 0.3-fold or less by endotoxin in wild-type mice, but was not decreased by endotoxin in NOS2−/− mice. (DOCX 2071 kb) [file 12931_2019_1062_MOESM2_ESM.docx]

**Additional file 3: Table S3**


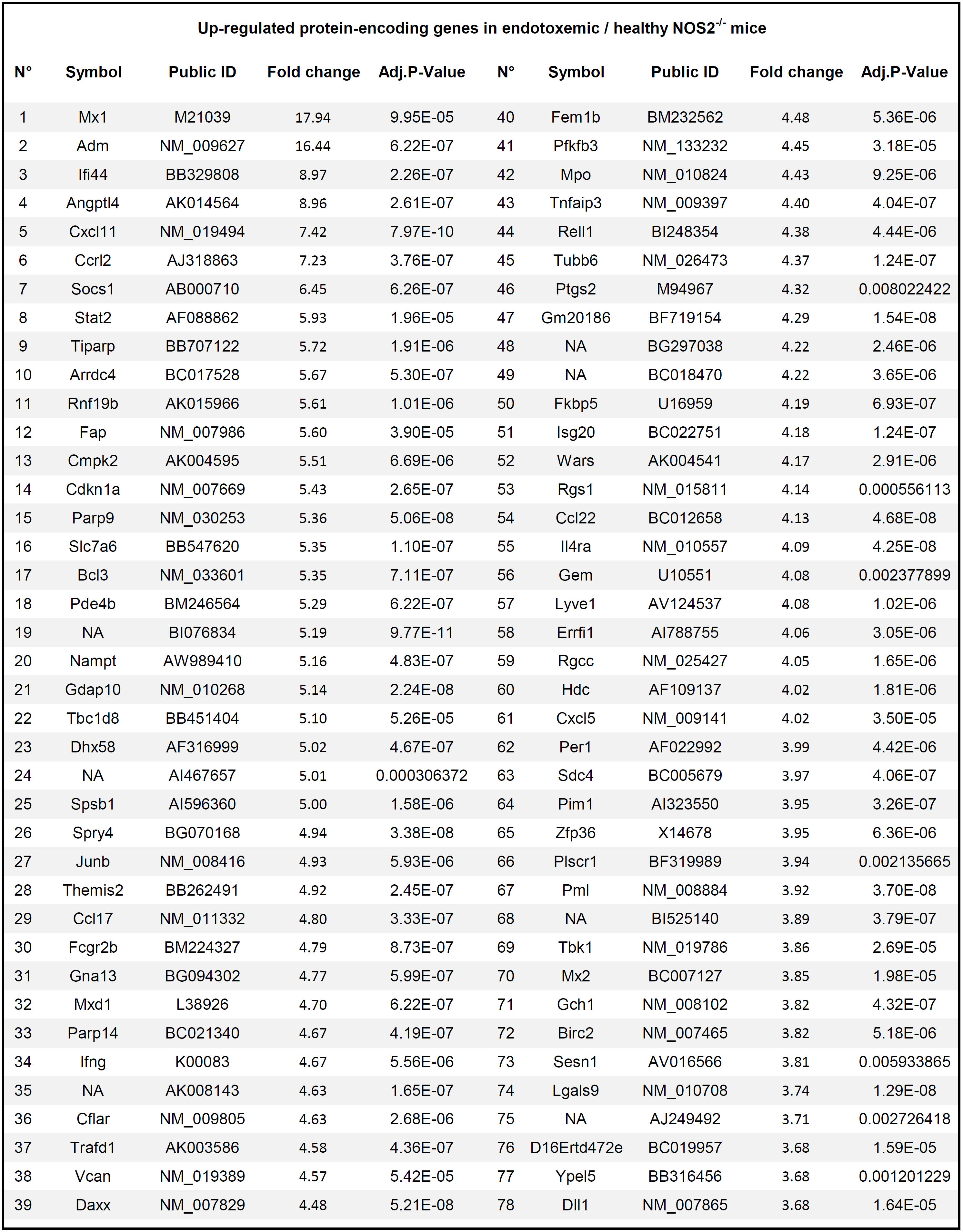


**SUPPLEMENT TABLE 3 (continued)**


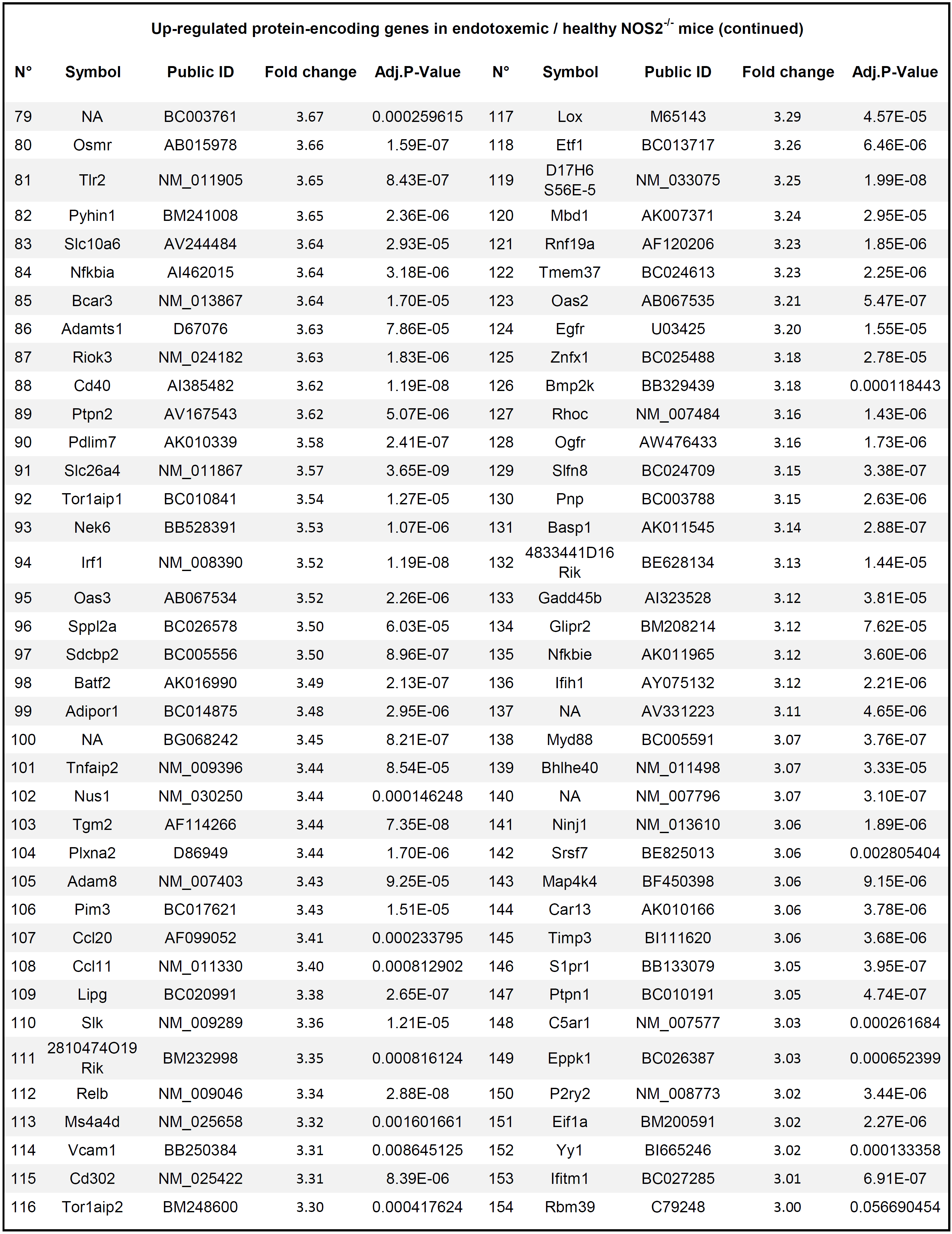

Supplement: Supplementary file 3 — Up-regulated genes in the lungs of endotoxemic NOS2−/− mice, but not in endotoxemic wild-type mice. Expression of 155 genes was increased more than three-fold by endotoxin in NOS2−/− mice, but was not similarly increased by endotoxin in wild-type mice. (DOCX 3039 kb) [file 12931_2019_1062_MOESM3_ESM.docx]

**Additional file 4: Table S4**


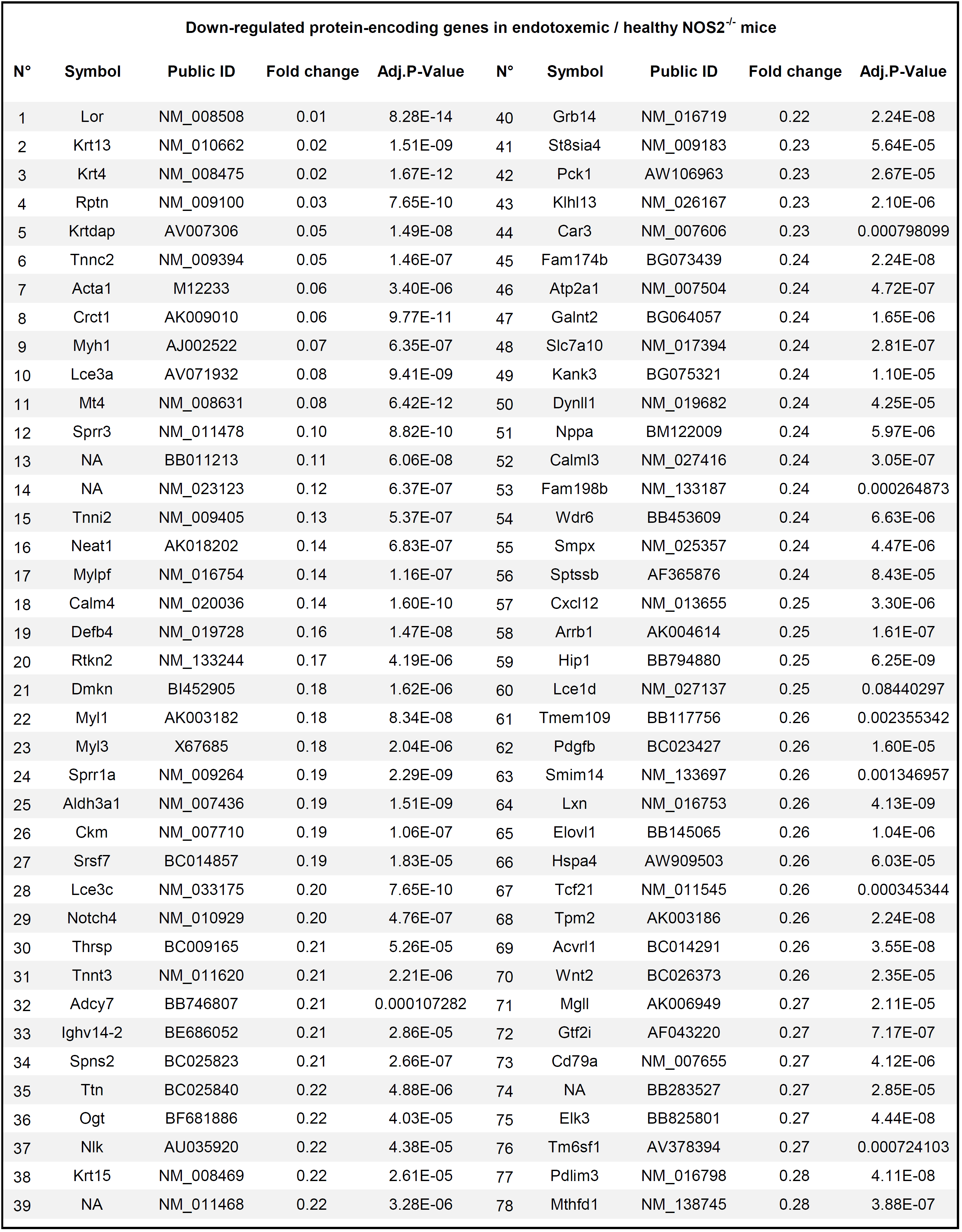


**SUPPLEMENT TABLE 4 (continued)**

Supplement: Supplementary file 4 — Down-regulated genes in the lungs of endotoxemic NOS2−/− mice, but not in endotoxemic wild-type mice. Ninety-three genes were decreased to 0.3-fold or less by endotoxin in NOS2−/− mice, but were not decreased by endotoxin in wild-type mice. (DOCX 2346 kb) [file 12931_2019_1062_MOESM4_ESM.docx]
